# Deep Learning-Based Visual Complexity Analysis of Electroencephalography Time-Frequency Images: Can It Localize the Epileptogenic Zone in the Brain?

**DOI:** 10.3390/a16120567

**Published:** 2023-12-15

**Authors:** Navaneethakrishna Makaram, Sarvagya Gupta, Matthew Pesce, Jeffrey Bolton, Scellig Stone, Daniel Haehn, Marc Pomplun, Christos Papadelis, Phillip Pearl, Alexander Rotenberg, Patricia Ellen Grant, Eleonora Tamilia

**Affiliations:** 1 Fetal-Neonatal Neuroimaging and Developmental Science Center, Division of Newborn Medicine, Department of Medicine, Boston Children’s Hospital, Harvard Medical School, Boston, MA 02115, USA; 2 Department of Neurology, Boston Children’s Hospital, Harvard Medical School, Boston, MA 02115, USA; 3 Division of Epilepsy Surgery, Department of Neurosurgery, Boston Children’s Hospital, Harvard Medical School, Boston, MA 02115, USA; 4 Department of Computer Science, University of Massachusetts Boston, Boston, MA 02115, USA; 5 Jane and John Justin Institute for Mind Health, Cook Children’s Health Care System, Fort Worth, TX 76104, USA

**Keywords:** epilepsy, deep learning, visual complexity, machine learning, image analysis, intracranial EEG, neurosurgery, MRI

## Abstract

In drug-resistant epilepsy, a visual inspection of intracranial electroencephalography (iEEG) signals is often needed to localize the epileptogenic zone (EZ) and guide neurosurgery. The visual assessment of iEEG time-frequency (TF) images is an alternative to signal inspection, but subtle variations may escape the human eye. Here, we propose a deep learning-based metric of visual complexity to interpret TF images extracted from iEEG data and aim to assess its ability to identify the EZ in the brain. We analyzed interictal iEEG data from 1928 contacts recorded from 20 children with drug-resistant epilepsy who became seizure-free after neurosurgery. We localized each iEEG contact in the MRI, created TF images (1–70 Hz) for each contact, and used a pre-trained VGG16 network to measure their visual complexity by extracting unsupervised activation energy (UAE) from 13 convolutional layers. We identified points of interest in the brain using the UAE values via patient- and layer-specific thresholds (based on extreme value distribution) and using a support vector machine classifier. Results show that contacts inside the seizure onset zone exhibit lower UAE than outside, with larger differences in deep layers (L10, L12, and L13: *p* < 0.001). Furthermore, the points of interest identified using the support vector machine, localized the EZ with 7 mm accuracy. In conclusion, we presented a pre-surgical computerized tool that facilitates the EZ localization in the patient’s MRI without requiring long-term iEEG inspection.

## 1. Introduction

Epilepsy is a neurological disorder that affects about 1–2% of the world population. About 1/3 of these patients do not respond to anti-seizure medication and continue to have frequent seizures. This is called drug-resistant epilepsy, and these patients are generally referred for surgical resection of the brain region that is responsible for generating the seizures (named the epileptogenic zone, EZ). This area can be only estimated indirectly through a variety of diagnostic tools that allow defining different cortical zones, each of which is an estimate of the EZ. Firstly, non-invasive electrophysiological tests are performed to estimate the EZ, which include the conventional EEG, high-density EEG, magnetoencephalography, magnetic resonance imaging (MRI), functional MRI, positron emission tomography, and single photon emission computed tomography. The results of these non-invasive tests can be insufficiently concordant and fail to derive a clear hypothesis about the location of the EZ in many children (more often than in adults primarily because the syndromes amenable to surgery are much more heterogeneous in pediatric epilepsy). In these cases, long-term monitoring with intracranial EEG (iEEG) is needed in order to evaluate the possibility of surgery and/or delineate the exact location and extent of the EZ. Direct exploration of the brain with iEEG over several days is, to date, the gold standard to capture seizures and pinpoint the origin of the seizures in the brain, also named the seizure onset zone (SOZ), which represents the best estimator of the EZ. The SOZ identification is typically performed by reviewing ictal data (recorded during seizures), yet seizures are unpredictable; thus, the majority of the recorded iEEG data correspond to the resting state (or absence of a seizure), which is termed an interictal state. This suggests that the development of reliable, interictal methods to estimate the SOZ electrodes without depending on the occurrence of seizures is essential. Interictal data contain vast amounts of information regarding the underlying pathology and can be explored to estimate the EZ and provide surgical guidance. One of the most traditional interictal markers on the EEG is the interictal epileptic discharges, also named interictal spikes, which are seen in the 1–70 Hz frequency range. Although several deep learning approaches (convolutional neural network-based algorithms) have been developed to identify interictal epileptic discharges on the iEEG signals in the time domain [1], the human reading and marking remain the gold standard in clinical practice to identify these signal patterns in the time domain. Reviewing interictal data, though, is extremely time demanding and challenging to human readers who have to look at days of iEEG recording from numerous electrodes in order to identify the contacts showing epileptiform activity.

Visual scrutiny of the iEEG time-frequency (TF) images can be an alternative way to review iEEG signals, allowing a detailed inspection in both the time and frequency domain. Yet, this can be arduous for the human reader: subtle features of the TF image may be interictal indicators of the EZ that are not perceptible by the human eye. For this reason, the development of novel algorithms and computerized tools to interpret interictal iEEG TF images may be extremely useful in locating the EZ in the brains of patients with drug-resistant epilepsy.

### 1.1. TF Image Analysis in Epilepsy

Several algorithms for the analysis of iEEG signals in the time, frequency, and TF domain have been proposed for localizing the EZ. TF approaches are based on a time-compressed visual representation of the EEG signal. These TF images are sensitive and efficient screening tools that have been mostly used by expert reviewers to analyze ictal signals for the identification of seizures [2]. In the context of seizure detection, these spectrogram patterns (or TF images) can be classified as solid flames, irregular flames, broadband monotonous, narrowband monotonous, stripes, low power, and artifacts [2]. Based on these guidelines, clinicians are able to identify seizures with a sensitivity ranging between 70% and 90% (estimates based on several studies) [3–10].

In patients implanted with iEEG prior to surgery, the visual inspection of the TF images is time consuming (up to about 150 iEEG electrodes may be placed in the brain, and thus up to 150 images must be visually inspected) and subjective to interobserver variability. The level of expertise of the neurophysiologist greatly influences decision making. Algorithms have been proposed to automate the process of reading the iEEG signal as a TF image using advanced signal and image processing techniques. Most studies have focused on the ictal or the pre/post-ictal interval to locate the EZ. An index was proposed by Bartolami et al. (2008) to predict the EZ [11] by combining the time and frequency information in the signal to assess the seizure dynamics. In other studies, TF images were able to characterize the suppression of beta and increase in gamma activity at the SOZ [12] or to predict the area that was removed during surgery [11–13]. Studies have also reported that TF patterns can be used to identify the underlying pathology causing seizures [14–16]. Several studies have also looked at the transition to seizure periods and compared them with the interictal regions using TF images.

### 1.2. Deep Learning for the Analysis of Visual Complexity

Visual complexity is commonly defined as the extent of intricacies and details inherent in an image or the cognitive challenge associated with the observation and description of said image. It may also be defined by the informational content encapsulated in the image and the principles governing its organization.

Deep learning algorithms are largely used in computer vision research [17–24]. One recent advance in this direction was presented by Saraee et al. [25], who focused on quantifying visual complexity, an image attribute that human operators can subjectively evaluate. Unlike other black box-based approaches, this method results in a quantitative metric of visual complexity that correlates with human perception. The authors used a pre-trained VGG net and extracted the unsupervised activation energy (UAE) from the network layers, demonstrating that it carries information about the image complexity that is meaningful to people [25].

In the present study, we used the Saraee et al. deep learning-based tool [25] to quantify the visual complexity of iEEG TF images and tested, for the first time, the effectiveness of this method in the identification of the EZ in the patient’s brain when combined with thresholding and machine learning algorithms for the identification of the electrodes of interest.

### 1.3. Our Contribution

In this work, we propose the use of a deep learning-based metric of visual complexity for the interpretation of iEEG TF images extracted from different points in the brain of patients with drug-resistant epilepsy. As iEEG electrodes can be accurately localized within the patient’s brain MRI, we combined the deep learning visual complexity analysis with thresholding and machine learning algorithms to identify the electrodes (or points) of interest in the patient’s brain.

We hypothesize that (i) the electrodes recording from the epileptogenic brain tissue have lower TF visual complexity as compared to others, as this reflects underlying epileptiform activity rather than the chaotic background, and (ii) that the UAE of the iEEG TF images can be used to determine the brain area to resect on the patient’s MRI.

The ultimate goal of this study is to present a pre-surgical computerized tool that is able to facilitate the localization of the EZ in the patient’s MRI space and help surgical planning using brief interictal iEEG without requiring long-term ictal recordings. To this purpose, we analyzed data from 20 children who underwent iEEG recording before surgical resection or ablation of the EZ in their brains. We computed the TF images from brief interictal iEEG signals recorded from all the implanted electrodes in their brain and studied how the TF image visual complexity varied in relation to the EZ. We also used thresholding and machine learning algorithms to identify the electrodes of interest based on their UAE values and estimated their localization error (distance from the EZ).

## 2. Materials and Methods

### 2.1. Patient Cohort

We analyzed the iEEG data of 20 pediatric patients with drug-resistant epilepsy who underwent resective surgery after extra-operative iEEG at the Epilepsy Center of Boston Children’s Hospital. A total of 1928 iEEG contacts were analyzed, with a mean of 96 contacts per patient. We selected patients who obtained seizure freedom for at least 12 months after surgery (Engel scale 1) and for whom we had post-implantation CT scans and pre- and post-operative MRIs. The study protocol received approval from the Boston Children’s Hospital Institutional Review Board (IRB-P00035192), which waived the need for written informed consent as the study was retrospective and posed minimal risk to patients.

### 2.2. iEEG Acquisition and Data Selection

The iEEG was recorded with subdural grids and strips and/or depth electrodes. Data were recorded using XLTEK NeuroWorks (Natus Inc., Golden, CO, USA). As part of our institution’s clinical practice, pediatric epileptologists review each patient’s iEEG on a daily basis and extract multiple 5 min segments that contain considerable interictal epileptiform activity [26–28]. We retrospectively reviewed these segments and selected the first 2 min of data for each patient without any major artifacts, technical disruptions, or clinical events marked on EEG annotations. Channels with continuous artifacts were excluded.

### 2.3. Localization and Classification of Contacts

**Figure 1.**
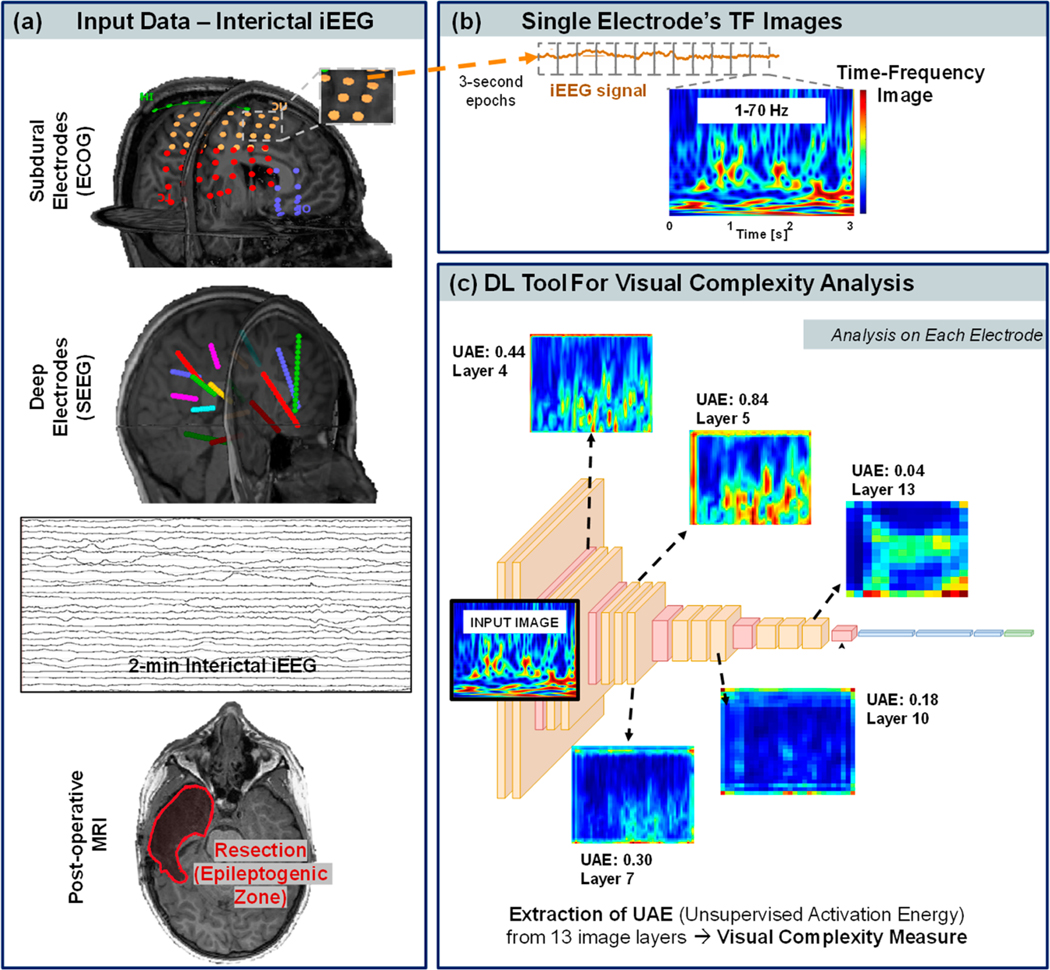
Methodological pipeline. (**a**) Input intracranial EEG (iEEG) data. Different colors correspond to different grids or depth electrodes. (**b**) Time-frequency (TF) images extracted from each iEEG contact in three frequency ranges (TF analysis was performed every 3 s). (**c**) Design of the deep learning (DL) tool that was used to extract values of visual complexity from each TF image. The input image is deconstructed into 13 layers (13 different images). For each layer of the image, we computed the unsupervised activation energy (UAE), which is a measure of the image’s visual complexity.

We determined the anatomical location of iEEG contacts in the patient’s pre-operative MRI space (see Figure 1a) by co-registering the post-implantation CT with the pre-operative MRI using statistical parametric mapping [29] within the Brainstorm software (April 2022) [30]. Each contact coordinate was determined on the co-registered CT-MRI image. To account for the brain shift that occurs after electrocorticographic implantation [31], subdural electrodes were projected onto the cortical surface (reconstructed via FreeSurfer). When both subdural and depth electrodes were implanted, depth electrodes were also adjusted to compensate for brain shift [31].

### 2.4. Epileptogenic Zone (EZ), Seizure Onset Zone (SOZ), and Post-Surgical Outcome

The EZ is the area of the brain that is indispensable for the generation of epileptic seizures [32]. It is a theoretical concept, meaning that it cannot be directly measured but only deduced postoperatively: if the patient is seizure-free after surgery, we conclude that the EZ must have been included in the resected brain area [32]. Thus, in our cohort, we defined the EZ as the area of the brain that was resected during the surgery (see Figure 1a) since all the patients became seizure free postoperatively. To segment the resection cavity in the patient’s brain, we co-registered pre-surgical and post-surgical MRI and used the MATLAB-based app developed by our group to outline the resection [33,34], followed by manual correction (3D Slicer software, v4) when needed. We measured the distance of each iEEG contact from the EZ as the Euclidean distance of the contact center from the closest resection margin. We then labeled each iEEG contact as within the EZ when having a distance below 5 mm (to account for post-operative brain shift and co-registration inaccuracies due to the child’s brain growth).

The SOZ is the area of the cortex from which clinical seizures are actually generated, as opposed to the EZ, which is the area of the cortex that is indispensable for seizure generation. Therefore, the SOZ can be actually measured during the pre-surgical evaluation in order to obtain an estimate of the EZ. At our institution, the SOZ is determined during each patient’s long-term iEEG monitoring: independently from this study, pediatric epileptologists reviewed all the patients’ seizures throughout the recording period and identified the SOZ as the iEEG contact/s showing the earliest change in activity associated with clinical or subclinical seizures [35,36]. We thus defined the SOZ in the same way for this study, based on the patient’s clinical records. Although the SOZ is regarded as the best estimate of the EZ, there may be parts of the SOZ that do not need to be resected to gain seizure freedom as well as parts of the EZ that are not measured as SOZ but are still able to generate seizures.

Based on the criteria above, 797 out of 1928 iEEG contacts (41%) were labeled as within the EZ (mean: 40 contacts per patient), while 285 contacts (15%) were labeled as within the SOZ (mean: 14 contacts per patient). The post-surgical outcome was evaluated by a board-certified pediatric epileptologist (J.B.) from the most recent follow-up visit using the Engel scale [37].

### 2.5. Time-Frequency Representation

We filtered the signal of each iEEG contact with a Kaiser window-based finite impulse response bandpass filter of 1–70 Hz. As Figure 1b shows, each iEEG signal was then segmented into 3 s epochs, and the Morlet wavelet transform was used to convert the time-domain signal into a time-dependent spectrum. Using the wavelet transform, the signal is convolved with various filter kernels of specific characteristics called wavelets, resulting in a spectrum with amplitude and phase. There are several families of wavelets, each representing a specific frequency band. The general equation for signal decomposition using wavelets is given by Equation (1).

(1) $$S_{w}(f,t)=s(t)*w_{f}(t)$$

where $w_{f}(t)$ is the wavelet, s(t) is the input signal, and ∗ represents convolution. The Morlet wavelet is commonly used in neuroscience for EEG signal analysis [38–40]. The envelope of this wavelet is a Gaussian.


(2) $$W^{w}(t,f)=exp\left(-\frac{t^{2}}{2\sigma^{2}}\right)\cdot exp(i2\pi ft)$$

In order to preserve the time and frequency resolution when the data are fed to the following neural network for visual complexity analysis (see next section), we fixed the time resolution to 8.9 ms and the frequency resolution to 0.30 Hz. This resulted in an image with a dimension of 224 × 224. The spectrum was normalized to a range of [0, 1] and eliminated any effects of power differences across contacts and between subjects. This was then coded into RGB space using a jet color map (Figure 1b) as it uses all three-color channels, unlike incremental color maps, which mainly use a particular color channel. The resultant 224 × 224 × 3 spectrum was used for the following deep learning-based analysis of the image’s visual complexity.

**Figure 2.**
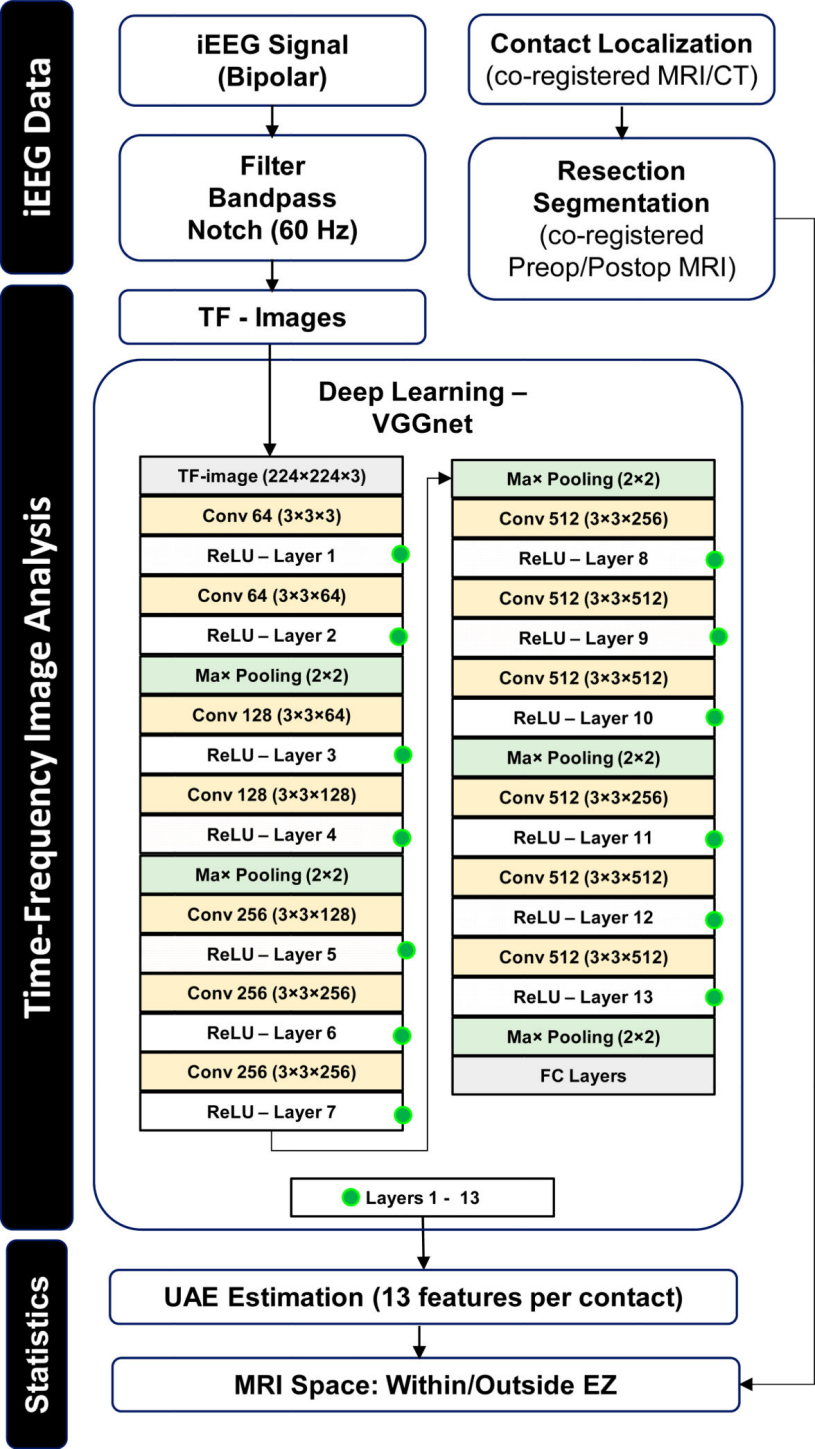
Methodological flowchart. Schematic outline of the iEEG signal processing, the TF image analysis with the deep learning (VGGnet) architecture (where conv represents the convolution layer; ReLU is the rectified linear unit, and FC the fully connected layers), and the point-of-interest identification. POI stays for “Point Of Interest”, and SVM for “support vector machine”.

Figure 2 schematizes the whole methodological workflow we propose in this study to analyze brief interictal iEEG data.

### 2.6. Deep Learning-Based Visual Complexity

We used the deep learning-based visual complexity assessment tool developed by Saraee et al. [25] to analyze images with objects and scenes, where the scores of visual complexities of images (in seven categories) were crowdsourced and compared to the output of the neural network. Here, we built on that work and used their tool to study the TF image complexity of each iEEG signal. We used an unsupervised deep learning tool based on a pre-trained VGG16 (Visual Geometry Group) model consisting of 16 layers, which is outlined in Figure 1c and in the Figure 2 flowchart. The literature suggests that the UAE values obtained from the VGG16 architecture linearly correlate with the human-scored visual complexity when tested on scenes from the SAVOIAS dataset [25,41–43]. Each layer of the network is activated based on the differences in the image properties, such as texture, shape, and edges. We extracted the UAE from each of the 13 convolutional layers to quantify their visual complexity. This results in 13 UAE values, or visual complexity features, per iEEG contact (where the average UAE value across all the 3 s epochs was used).

The UAE is defined for each layer as the average of the overall values of receptive fields in all the layer channels:

(3) $$\text{UAE}(L)=\frac{1}{h*w*d}\sum_{i,j,k=1}^{h,w,d}F_{L}[i,j,k]$$

where L is the layer, F is the feature map, h is the height of the feature map, w is the width of the feature map, and d is the depth (number of deep learning channels) of the feature map, respectively. We extracted the feature maps after the rectified linear units (or RELU) to ensure a positive UAE value, where the larger the UAE, the more complex the TF image.

### 2.7. Identification of the “Points of Interest” Based on Their TF Image Visual Complexity

**Figure 3.**
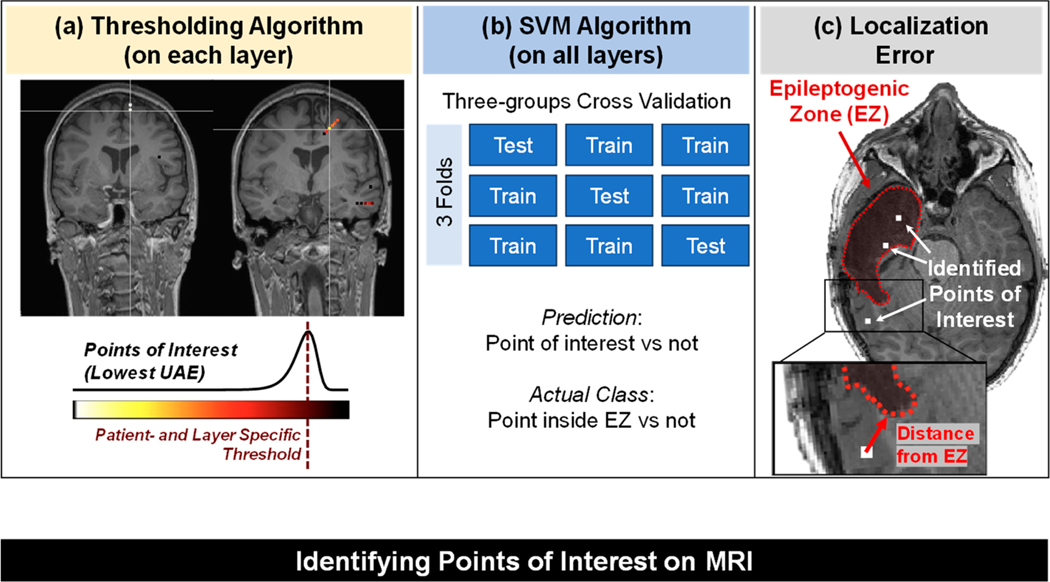
Identifying points of interest (POIs) on the patient’s MRI.

We employed two different algorithms to identify the “points of interest” in the patient MRI space based on the UAE values of the iEEG TF images, which are also outlined in Figure 3:

1. Patient- and layer-specific thresholding algorithm: thresholds were identified by fitting each layer’s UAE values to an extreme-value distribution [44–46]. The tail of the distribution contains the contacts with lower complexity, which we regarded as our points of interest in the brain. Extreme value theory pertains to the science of modeling and quantifying occurrences that have extremely low probabilities. Extreme value distribution can be generalized to distributions with the following probability density function (p) that depends on the location parameter (μ) and scale parameter (σ).
(4) $$p=\sigma^{-1}exp\left(\frac{x-\mu }{\sigma }\right)exp\left(-exp\left(\frac{x-\mu }{\sigma }\right)\right)$$
  We fit the UAE values to the extreme value distribution to obtain the probability density function and then identified our points of interest (the tail of the distribution) as those with UAE values lower than the distribution’s location parameter (Figure 3a);
2. We used the UAE values from all the layers and passed them to a support vector machine’s classifier (see Figure 3b) with a linear kernel to classify each contact as a point of interest or not, where the ground truth label consisted of whether a point (contact) was inside or outside the EZ. As a validation strategy, we adopted a three-fold cross-validation, where we split the subjects into three distinct groups with similar class distribution [47] (similar proportion of contacts inside vs. outside the EZ). The number of contacts in the two classes (inside versus outside the EZ) for each group was: for Group 1, 259 inside versus 375 outside; for Group 2, 294 versus 378; and for Group 3, 244 versus 378. The groups were defined such that the subjects in each group were distinct (number of subjects per group: 7 in G1, 7 in G2, and 6 in G3).

To quantify the accuracy of our algorithms in localizing the EZ, we computed the average Euclidean distance between the identified points of interest and the closest EZ margin, as schematized in Figure 3c. This provided an estimate of the localizing capabilities of our algorithms within the context of an epilepsy surgery application.

### 2.8. Statistical Analysis

Statistical analysis was performed in MATLAB. We first tested whether the iEEG contacts recorded from the SOZ have different UAE values compared to those recorded from brain regions that are not part of the SOZ (thus regarded as non-epileptogenic tissue). To this purpose, we compared the mean UAE of the contacts inside and outside the SOZ (Wilcoxon signed rank test) for each layer.

Furthermore, we assessed the ability of our TF complexity method to locate the EZ in the patient’s MRI by quantifying the distance of our points of interest from the EZ and comparing this (Wilcoxon signed rank test) with the distance of the other iEEG contacts (not of interest).

We considered *p*-values ≤ 0.05 significant. Results are reported as median (inter-quartile range, IQR).

## 3. Results

We analyzed data from 20 patients (6 females) with a mean age at the time of the iEEG of 12 ± 6 years. In 16 patients, the EZ was in the left hemisphere; the resected EZ was in the temporal lobe in seven patients, in the frontal lobe in eight patients, and in the parietal, occipital, frontotemporal or multiple lobes in the remaining five patients. Two patients had unknown epilepsy etiology without a visible lesion on the pre-operative MRI.

### 3.1. UAE Values and Their Correspondance to the Seizure Onset Zone

**Figure 4.**
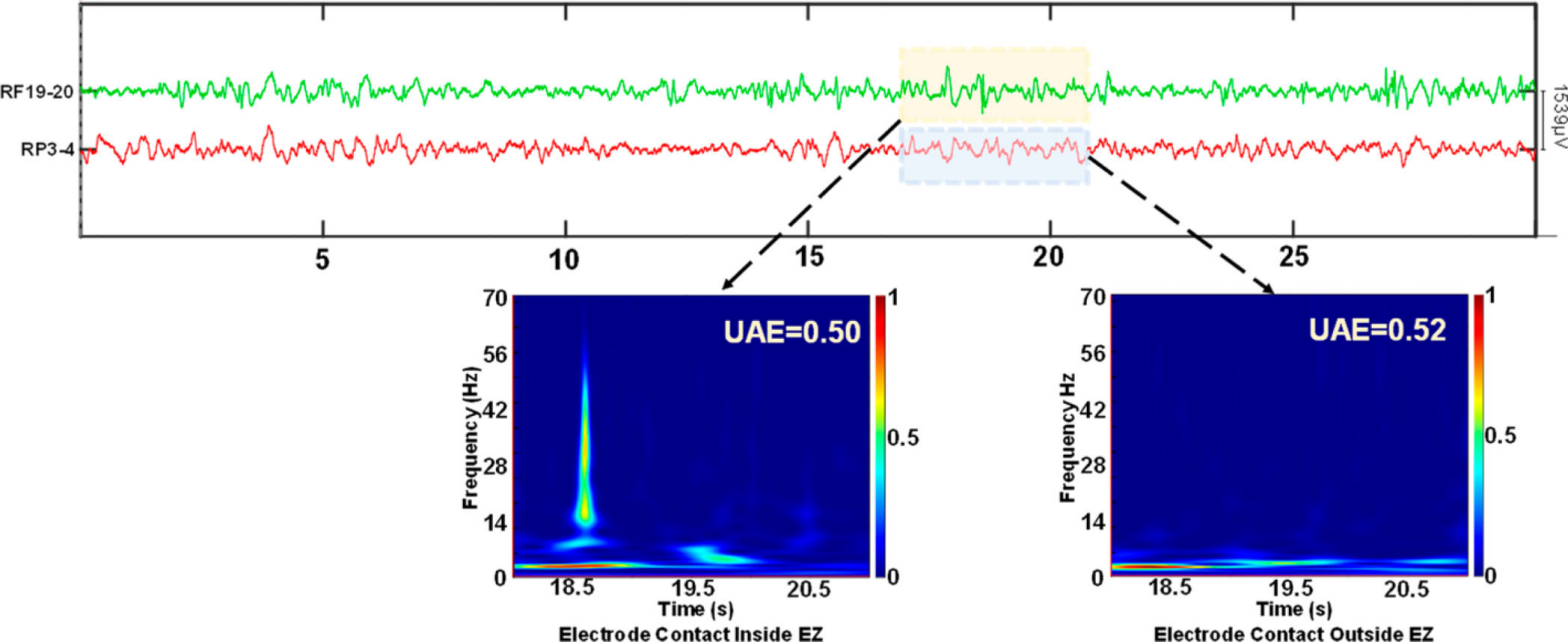
iEEG signals from a patient with seizures originating in the frontal lobe. Two representative signals, one from the frontal lobe (inside EZ) and another from the parietal lobe (outside EZ), and their corresponding TF image.

We generated the TF images of 1928 iEEG signals (mean: 96 signals per patient) and compared their 13 features of visual complexity between images generated from inside the SOZ and those generated from outside the SOZ. Figure 4 shows two iEEG signals from a patient, where the green signal is recorded from a contact located inside the EZ (or resection) and SOZ, while the red signal is recorded from healthy brain tissue (non-SOZ contact). The TF image of the EZ signal shows a strong wideband frequency component above 10 Hz, which is often characteristic of the presence of an epileptiform sharp discharge in the signal and makes the TF image of “easy visual interpretation” (and low complexity). For the other contact, the TF spectrum is concentrated in the lower frequency bands, while the rest of the image (frequencies above 6 Hz) presents very subtle differences, which make it difficult to determine each frequency contribution, thereby leading to a “harder visual interpretation” and thus higher visual complexity, reflected in the higher UAE.

**Figure 5.**
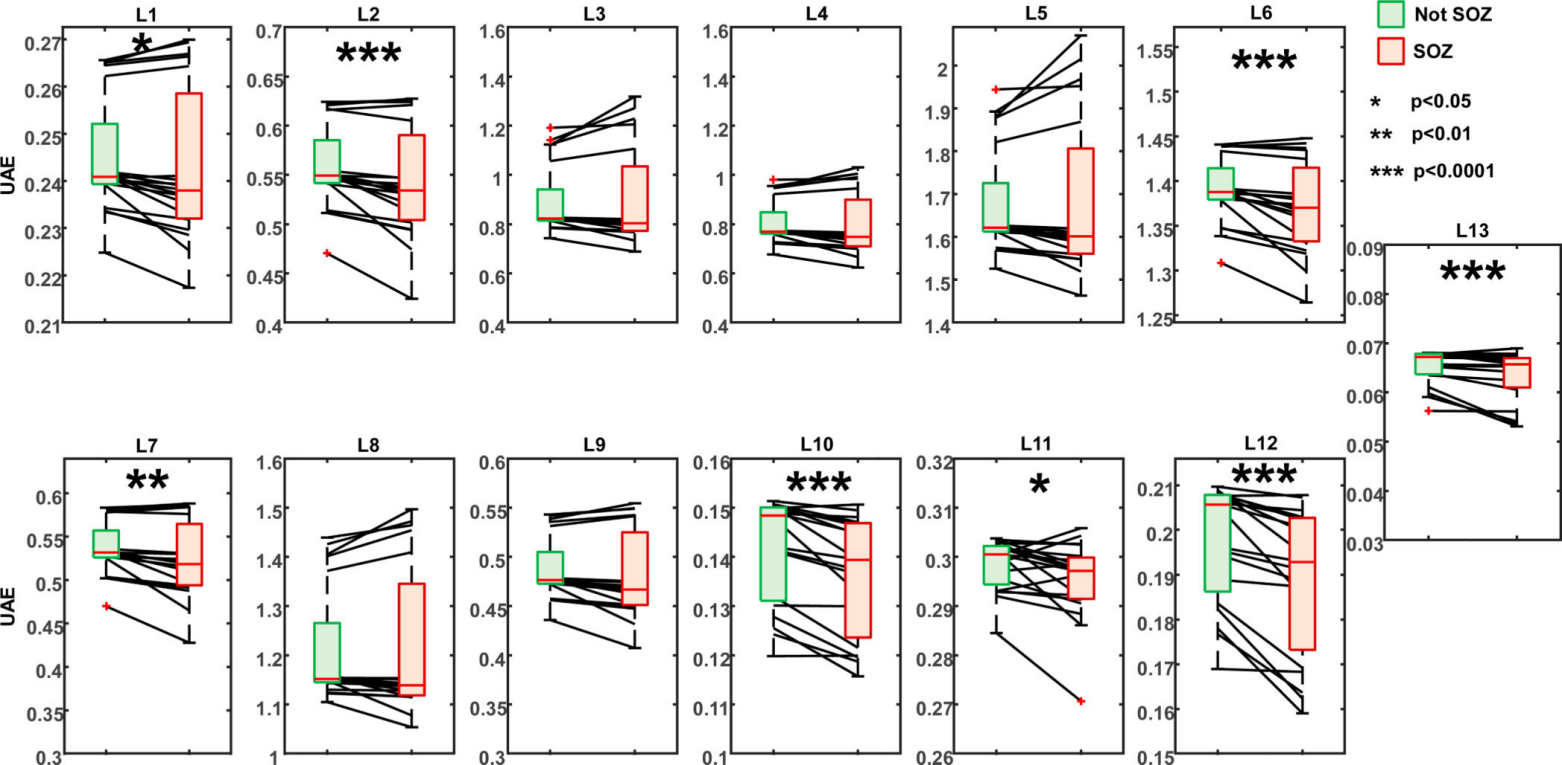
UAE values inside the SOZ vs. outside the SOZ across the various layers of the VGGnet. The whiskers of the boxplots extend to the most extreme data points not considered outliers (product of 1.5 and the inter-quartile range), while the outliers are plotted individually using the red ‘+’ marker symbol.

When performing statistical analysis across the whole cohort of patients between contacts inside and outside the SOZ, this difference in UAE values was confirmed, as shown in Figure 5. We found that the mean UAE values inside the SOZ were lower than outside the SOZ when looking at various layers (*p* < 0.05 in layers 1 and 11; *p* < 0.01 in layer 7; and *p* < 0.0001 in layers 2, 6, 7, 10, 12, and 13). The largest difference was found in layer 12 (*p* = 0.00016, Cohens D = 0.5, C.I.: 1.18–0.136), followed by layer 10 (*p* = 0.00029, Cohens D = 0.41, C.I. = 0.97–0.11). The other layers, namely layers 2, 6, and 13, showed Cohens D values of 0.30 (C.I. = 0.79–0.01, *p* = 0.00097), 0.43 (C.I. = 1.085–0.04, *p* = 0.00046), and 0.35 (C.I. = 0.98–0.06, *p* = 0.00097), respectively. Upon correcting for multiple comparisons using Bonferroni correction, statistically significant differences are still seen in layers 2 and 13 (*p* < 0.05) and layers 6, 10, and 12 (*p* < 0.01).

### 3.2. Identification of the “Points of Interest”

**Figure 6.**
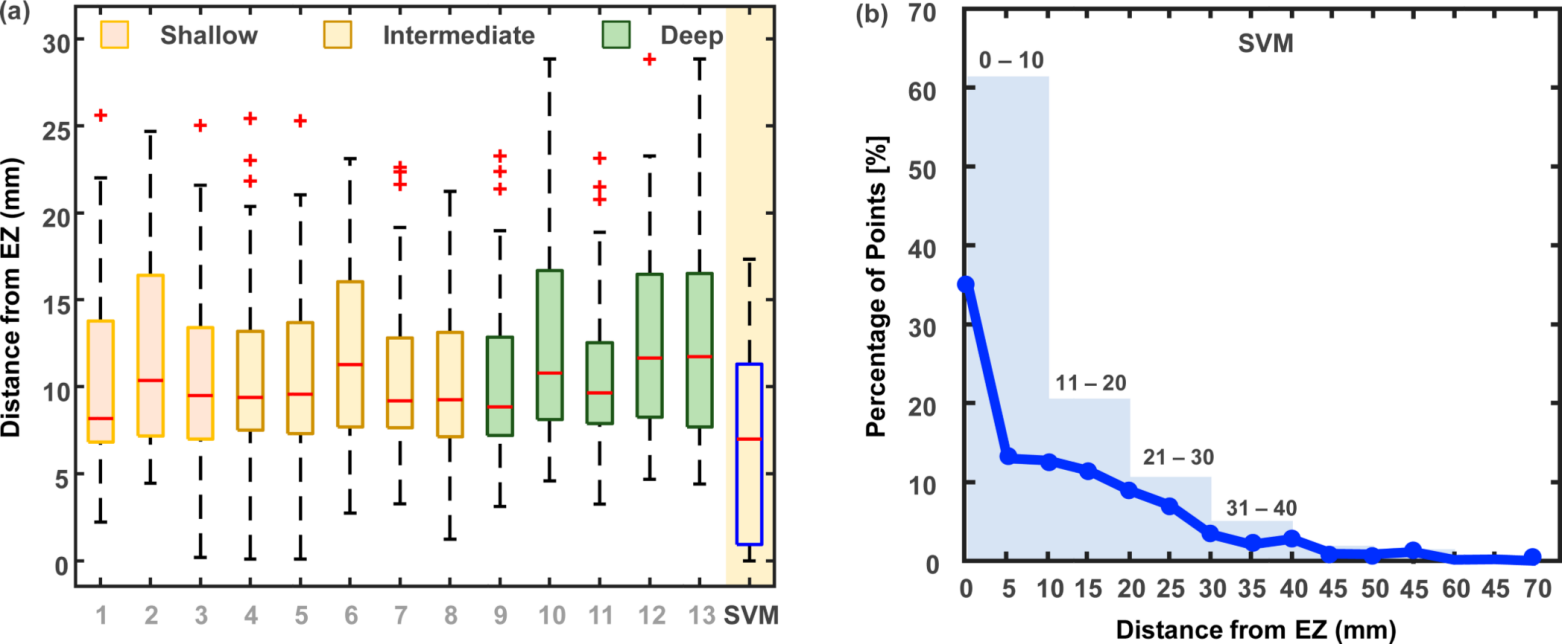
(**a**) Points of Interest and their proximity to the EZ when using each individual layer or the support vector machine (SVM) model and (**b**) the percentage of points of interest estimated using SVM and their proximity to the EZ. The whiskers of the boxplots extend to the most extreme data points not considered outliers (product of 1.5 and the inter-quartile range), while the outliers are plotted individually using the red ‘+’ marker symbol.

Figure 6a shows the proximity to the EZ of the points of interest identified through the extreme value distribution-based algorithm applied to the various investigated layers of the TF image as well as through the support vector machine’s classifier used on all the layers. We can observe that the individual layers are able to identify the EZ within median proximity of 8–12 mm (Layer 1: 8.17, Layer 2: 10.36, Layer 3: 9.50, Layer 4: 9.40, Layer 5: 9.57, Layer 6: 11.27, Layer 7: 9.19, Layer 8: 9.26, Layer 9: 8.85, Layer 10: 10.79, Layer 11: 9.66, Layer 12: 11.63, and Layer 13: 11.73).

When we combine features from all layers and use a support vector machine’s classifier-based classifier, we observe that the points of interest are very proximal to the EZ with a median distance of 7 mm, which is significantly shorter compared to using any individual layer threshold (support vector machines classifier vs. Layer 1, 3, 4, 5, and 12: *p* < 0.001; support vector machines classifier vs. layer rest of the layers: *p* < 0.05).

Figure 6b shows the distribution of points of interest across proximity to the EZ. About 35% of the points are within the EZ (indicated by the blue line), and about 60% of them are inside a 10 mm radius (indicated by the barplot).

We tested the effect of the type of pathology on the localizing error and found that patients with focal cortical dysplasia (*n* = 8) have a lower localization error as compared to the other groups, which included unknown etiology, tumor, and polymicrogyria (6 mm vs. 12 mm; *p* = 0.018). We also tested the effect of age at surgery on localization error and found no significant correlation (*p* > 0.05).

## 4. Discussion

To our knowledge, this study is the first to test the use of a computer-vision deep learning tool for interpreting interictal iEEG TF images and the localization of epileptogenic points of interest within the brain of children with drug-resistant epilepsy. As outlined in the flowchart in Figure 2, we combined deep learning-based algorithms for the analysis of the image visual complexity with medical image co-registration techniques and thresholding and machine learning algorithms with the ultimate goal of identifying the epileptogenic points of interest in the patient’s brain MRI.

This study was driven by two main hypotheses that have been supported by our main findings: (1) the electrodes recording from the epileptogenic brain tissue reflect underlying epileptiform activity rather than chaotic background and thus have lower TF visual complexity as compared to others, and (2) the UAE of the iEEG TF images can help locate the EZ in the patient’s MRI.

Our first main finding is that the UAE values of the TF images extracted from the contacts inside the SOZ are significantly lower than those from non-SOZ contacts when using various (shallow, intermediate, and deep) layers of the deep learning network (see Figure 5). Several deep layers (layers 10, 12, and 13) exhibit strongly significant differences (*p* < 0.001), while these are seen less in the shallow layers, indicating that complex features (which quantify characteristics that are not easily interpretable by the human eye and are reflected by the deep layers’ UAE) play an important role in the differentiation of electrodes in EZ versus the healthy tissue. Interestingly, the deep layers exhibit comparatively lower UAE values (see Figure 5) than the shallow or intermediate layers. This might be due to the funneling nature of the neural network, where deeper layers have fewer weights. This result is particularly interesting if put into the context of the recent literature on deep learning visual complexity [25], which provides valuable insights into the correlation of the various layers with human perception. It is noteworthy that the first and last two layers of deep learning networks exhibit a lower correlation with the human perception scores compared to the intermediate layers (layers 4–8). The first layer primarily captures information related to edges and corners but lacks the high-level information required to evaluate visual complexity. On the other hand, the last layers are primarily tuned for classification tasks but do not correlate with the human assessment of visual complexity (as they capture highly complex features that may be unperceivable to the human naked eye). The intermediate layers, which encompass a combination of both low-level and high-level features, prove to be particularly correlated with human perception of visual complexity [25]. This literature adds depth to our understanding of how deep learning networks perceive and evaluate visual complexity and helps interpret our findings. Our data indicate that the deepest layers of the deep learning network are the most relevant when analyzing interictal iEEG TF images: this may suggest that the image characteristics that reflect underlying epileptogenicity are highly complex and not easily perceivable by the human reader. This further underscores the importance of a computerized tool that complements human perception and vision.

Our second key finding is that the points of interest we identified—based on their TF image’s UAE—presented a 7–12 mm accuracy in the localization of the EZ (see Figure 6), with the most accurate findings (7 mm) obtained when using a machine learning algorithm (SVM) to combine information from all the layers of the deep learning network (VGGnet). According to the Desikan–Killiany atlas map, the smallest area in the brain, such as the temporal pole, has an estimated radius of 6 mm, and a large area, such as the superior frontal gyrus, has an estimated radius of 30 mm. In addition, the typical gyral width of the para-hippocampal gyrus is between 11 and 21 mm [26,47,48]. Thus, an average localization error of 7 mm is very unlikely to cause a mis-localization at the anatomical level of the brain. Even though several studies have proposed novel methods to localize the EZ for epilepsy surgery planning, only a few of them have reported actual distance from the EZ (or resection) of the proposed methods. Recent works on localizing the EZ using electrical source imaging and/or iEEG resulted in errors of 5 mm [48] and 9 mm [49] or reported distances within 10 mm as concordant [50]. Our data thus suggest that the points of interest we identified on the patients’ MRI are concordant with the EZ and are comparable to the most recent results in epilepsy surgery.

The presented methodology shows great potential in the field of epilepsy care and the strategic planning for neurosurgery in individuals with drug-resistant epilepsy. The development of a computerized tool that uses deep learning-based visual complexity metrics to analyze interictal iEEG TF images represents a possible breakthrough (if further validated on larger cohorts) with the potential to significantly enhance the pre-surgical decision-making process and the effectiveness of surgeries in patients with drug-resistant epilepsy.

The proposed methods and algorithms overcome the longstanding challenges of relying on ictal data for EZ localization (where the challenge is due to the unpredictability of seizures). By introducing a multi-step approach to analyze interictal iEEG data, we propose a comprehensive automated solution for identifying iEEG electrodes with low TF visual complexity indicative of underlying brain epileptogenicity. This methodology has the potential to improve the pre-surgical planning when seizures are not captured and thus reduce the reliance on long-term ictal recordings.

In conclusion, the study’s innovative approach has the potential to improve drug-resistant epilepsy treatment, offering an accurate and automated means to localize the EZ in each patient’s MRI via brief iEEG data with an average localization accuracy of 7 mm. Further validation of the proposed method on a larger cohort could ultimately improve the pre-surgical evaluation of patients with drug-resistant epilepsy and enhance epilepsy surgery planning.

### 4.1. Comparison with Existing Methods

Existing methods for localizing the EZ in epilepsy surgery planning include localization of the SOZ via visual scrutiny of the patient’s ictal EEG data as well as localization of the irritative zone or high-frequency-oscillation zone via visual scrutiny and marking of interictal data. The approach for iEEG analysis proposed here is particularly advantageous when compared to such current methods since it provides a high localization accuracy through a fully automated analysis of very brief data as opposed to the SOZ estimation (which is highly dependent on seizure capturing and human interpretation) or to the irritative or high-frequency-oscillation zone estimation (which is also dependent on the human scrutiny of portions of data that are long enough to capture the patient’s representative spike or high-frequency-oscillation populations). Our innovative approach could complement existing methods, particularly in cases where the SOZ cannot be estimated (e.g., the patient does not seize during the monitoring, or their seizures are not of easy interpretation) or where the patient presents without any interictal discharges in the iEEG traces; in these cases, the iEEG would fail to derive a clear hypothesis about the location of the EZ, and thus alternative approaches would be extremely beneficial. Even though the proposed method reduces human effort, there are certain limitations associated with using this technique, which are not encountered when applying traditional clinical methods. Primarily, the deep learning method requires the conversion of the iEEG signal into time-frequency images, which require more space and require additional computational effort to load, process, and save. This means that additional efforts need to be taken for memory management, computational resource management, and garbage collection. Further, this method might be difficult to implement in long EEG recordings due to the above-mentioned complexities.

### 4.2. Considerations on the Automated Approach

Several researchers have used TF methods to characterize iEEG signals in epilepsy. They have found that the visual inspection of these images provides more information than traditional time series-based inspection. The study attempts to fully automate the visual inspection process by leveraging deep learning-based image inspection techniques. Even though this method reduces human effort, there are certain limitations associated with using this technique. Primarily, the deep learning method requires the conversion of the iEEG signals into TF images as input. These images require more space and additional computational effort to load, process, and save. This means that additional efforts need to be taken for memory management, computational resource management, and garbage collection. Further, this method might be difficult to implement in long-term EEG recordings due to the above-mentioned complexities.

### 4.3. Limitations and Future Directions

In terms of study limitations, we should acknowledge (i) the limited sample size, which hinders the generalizability of our current findings, and (ii) the heterogenicity of the pediatric cohort in terms of underlying pathology, which could potentially bias our results.

In the field of epilepsy, collecting large amounts of data is challenging as the time to add a patient with their surgical outcome would take a minimum of 1 year. Further, all patients diagnosed with epilepsy are not considered for surgery or for iEEG. We are continuously adding patients to our cohort as and when they become available. Multi-site collaborations are also warranted to increase sample size and generalizability. The other key issue that we need to address is the harmonization of data collection, as each of the centers has its own protocol for treating patients with epilepsy. We intend to test our hypothesis on a larger scale and test the feasibility of these features. This would enable us to test the generalizability of differences in pathology, age, sex, and race.

Our promising findings encourage further investigations on larger cohorts as these would allow to (i) control for potential confounding factors and (ii) train or tune the hyperparameters of the deep learning network on actual iEEG TF images (instead of utilizing pre-trained network architecture validated on generic real-world images). Our findings also warrant further investigations to compare the benefits of the proposed image-based method versus feeding the raw iEEG data into a machine learning or deep learning algorithm. Given that our current method mimics the human observer, we would like to leverage the power of modern signal processing and artificial intelligence to seek biomarkers suited to identify the pathology and work towards optimizing the resection volume to enhance the quality of life of patients with epilepsy. Finally, our data also open the avenues to exploring other applications of visual complexity metrics in EEG and medical imaging analysis.

The iEEG recordings present some challenges and limitations in clinical practice: they are costly, require a cooperative patient, carry surgical risks [51], and leave large brain areas unexplored. Thus, our promising findings prompt future efforts toward the translation of the proposed methodology to scalp EEG and magnetoencephalography data since they are non-invasive pre-surgical tools that can contribute to the pre-surgical estimation of the SOZ without the need for invasive recordings [26,28,52,53].

## Data Availability

Due to privacy and ethical restrictions, data will be made available upon request to the corresponding author.
